# The lesser the better? A systematic review and meta‐analysis of resection strategy in lung neuroendocrine tumors

**DOI:** 10.1111/jne.70176

**Published:** 2026-04-01

**Authors:** Gal Aviel, Ranin Hojerat, Islam Idais, Bruria Hirsh‐Raccah, Simona Grozinsky‐Glasberg, Anat Bel Ange, Oz M. Shapira, Amit Korach, Uzi Izhar, Ori Wald

**Affiliations:** ^1^ Department of Cardiothoracic Surgery Hadassah Medical Center and Faculty of Medicine, Hebrew University of Jerusalem Jerusalem Israel; ^2^ Department of Cardiology Hadassah Medical Center and Faculty of Medicine, Hebrew University of Jerusalem Jerusalem Israel; ^3^ Division of Clinical Pharmacy Institute for Drug Research, School of Pharmacy, Faculty of Medicine, Hebrew University of Jerusalem Jerusalem Israel; ^4^ Neuroendocrine Tumor Unit, ENETS Centre of Excellence, Division of Internal Medicine Hadassah Medical Center and Faculty of Medicine, Hebrew University of Jerusalem Jerusalem Israel

**Keywords:** lung neuroendocrine tumor, lobectomy, meta‐analysis, pulmonary carcinoids, sub‐lobar resection

## Abstract

**Introduction:**

Lung neuroendocrine tumors (LNETs) are rare, with surgical resection as the mainstay of treatment, although the optimal extent remains uncertain. Herein, we present the first meta‐analysis to assess the effect of resection extent (lobar vs. sub‐lobar) on overall survival.

**Methods:**

We conducted a systematic review of the literature to identify studies comparing overall survival following lobectomy versus sub‐lobar resection in LNETs. An inverse‐variance meta‐analysis was performed, and a Cox regression model was applied to reconstructed time‐to‐event data estimated from published Kaplan–Meier curves to generate pooled survival estimates.

**Results:**

Six studies encompassing 3,700 patients (lobectomy, *n* = 2,409; sub‐lobar resection, *n* = 1,291) were included in the final analysis. The pooled 5‐year overall survival for the entire cohort was 78.8% (95% CI, 76.6–81.1). No statistically significant difference in overall survival was observed between lobectomy and sub‐lobar resection (HR = 1.21; 95% CI, 0.80–1.83; *I* ^2^ = 0%). Segmentectomy and lobectomy demonstrated comparable survival (*p* = 0.38), whereas wedge resection was associated with higher mortality (HR = 2.02; 95% CI, 1.64–2.49; *I* ^2^ = 0%). Sampling of >10 lymph nodes was more frequent in lobectomy than sub‐lobar resection (29.1% [95% CI, 0.8–95.3] vs 7.4% [95% CI, 0.01–98], respectively), likely contributing to the higher rate of nodal pathologic upstaging observed in the lobectomy group (6.2% [95% CI, 0.2–64.9] vs 2.2% [95% CI, 0–99]).

**Conclusion:**

In this first meta‐analysis of surgical resection for LNETs, sub‐lobar resection and lobectomy showed no clear difference in overall survival. Adequate lymph node assessment remains essential, irrespective of the surgical approach.

## 1 INTRODUCTION

Lung neuroendocrine tumors (LNETs), also referred to as pulmonary carcinoids (PCs), are rare neoplasms with an annual incidence of 0.2–2 per 100,000 in the United States and Europe, accounting for 1–2% of primary lung cancers. Over the past two decades, incidence has increased, attributed to improved recognition, advances in diagnostic techniques, and a possible true rise in occurrence.^1^, ^2^

LNETs comprise well‐differentiated typical carcinoid (TC), which represents 85 to 90% of cases, and atypical carcinoid (AC), which accounts for the remainder. These tumors carry a relatively low burden of chromosomal alterations and infrequently harbor smoking‐associated TP53 mutations, in contrast to high‐grade lung neuroendocrine carcinomas.^3^ Consistent with their indolent biology, LNETs have a favorable prognosis, with 10‐year disease‐specific survival rates of 96% and 85% for stage I and II TC, and 88% and 75% for stage I and II AC, respectively.^4^

Accordingly, surgical resection is the mainstay of treatment for localized disease, with lobectomy as the traditional approach. Randomized trials in early‐stage non‐small‐cell lung cancer, a considerably more aggressive tumor than LNETs, have demonstrated that sub‐lobar resection is noninferior to lobectomy with respect to survival and oncologic outcomes,^5^, ^6^ raising the question of whether a similar strategy may be appropriate for selected patients with LNETs.

Prospective data comparing lobectomy with sub‐lobar resection in LNETs are lacking, and available retrospective studies have yielded inconsistent results,^7^ leaving the optimal surgical strategy uncertain. We therefore conducted a systematic review and meta‐analysis to compare overall survival following lobar versus sub‐lobar resection in patients with LNETs.

## 2 METHODS

We systematically searched Embase, the Cochrane Library, Google Scholar, and PubMed (MEDLINE) from database inception to September 26, 2024, in accordance with the *Cochrane Handbook for Systematic Reviews of Interventions*.^8^ The complete search strategy is provided in Table S2. Search strategies incorporated index terms, Medical Subject Headings (MeSH), and free‐text keywords related to the main concepts, without language or date restrictions. Eligible studies included randomized controlled trials (RCTs), cohort studies, and case–control studies comparing overall survival after sub‐lobar versus lobar resection in patients with typical or atypical pulmonary carcinoid tumors. We excluded duplicate publications, case reports, case series, pharmacokinetic studies in healthy subjects, reviews, expert opinions, editorials, letters, and comments. The systematic review and meta‐analysis were conducted in accordance with the *Preferred Reporting Items for Systematic Reviews and Meta‐Analyses* (PRISMA) guidelines for systematic reviews and meta‐analyses.^9^ The protocol was registered with the PROSPERO International Prospective Register of Systematic Reviews (CRD420251030224).

### 2.1 Data extraction and quality assessment

One reviewer (Gal Aviel) identified potentially eligible studies, and two reviewers (Ranin Hojerat, Islam Idais) independently assessed them for inclusion. Discrepancies were resolved by consensus or consultation with a third reviewer (Ori Wald). Reference lists of included articles were manually searched to identify additional studies. Data extraction was performed independently by Ranin Hojerat and Islam Idais. The quality of observational studies was assessed using the Newcastle–Ottawa Scale.^10^ For randomized controlled trials, we applied the Cochrane Risk of Bias tool and excluded those judged to be at high risk of bias.^11^ Observational studies with low Newcastle–Ottawa scores (<7) were not excluded a priori; however, a sensitivity analysis was conducted after their exclusion.

### 2.2 Data analysis

*Descriptive statistics*: The statistical analysis was performed using the *meta* package using RStudio (Version 4.2.2). The individual pooled weighted effects of baseline characteristics were calculated separately for the two patient groups (sub‐lobar and lobar resection). Pooled proportions were calculated using the *metaprop* function, and pooled means were calculated using the *metamean* function.

*Overall survival*: We used the calculated hazard ratios (HR) from each study in the *metagen* function using RStudio (Version 4.2.2). We implemented a random‐effects model with a Hartung‐Knapp adjustment to calculate the pooled HR and 95%‐confidence interval (CI). Tau^2^ was calculated using the Paule‐Mandel estimator. Inter‐study heterogeneity was evaluated using *I* ^2^ and Q. We performed a subgroup analysis according to the surgical strategy undertaken—sub‐lobar vs. lobar resection, segmentectomy vs. lobar and wedge resection vs. lobar.

*Sensitivity analyses*: We used the leaving‐one‐out method applied to the pooled HR according to the different subgroups, repeatedly omitting one study at a time and repeatedly calculating the HR and 95%‐CI.

*Study heterogeneity*: We calculated the *I* ^2^ and *Q* for each statistical test.

### 2.3 Publication bias

Assessment of publication bias was planned by visual inspection of the funnel plot, provided the analysis would include at least 10 studies.^12^

We applied the Grading of Recommendations Assessment, Development and Evaluation (GRADE) framework for meta‐analyses to evaluate the certainty of evidence.^13^ Each comparison and outcome was rated as high, moderate, low, or very low certainty based on the domains of risk of bias, inconsistency, indirectness, imprecision, and publication bias.

### 2.4 Pooled survival curves

Pooled survival curves were generated from approximated individual time‐to‐event data that were extracted from the published Kaplan–Meier survival curves. Briefly, we reconstructed the individual time‐to‐event data from Kaplan–Meier survival curves using the *KMfromIPD* package on R (Version 3.6.0).^14^ First, we separated the Kaplan–Meier graphs of each comparison using Adobe Illustrator. We subsequently exported the clean graphs for coordinate extraction using R. IPD reconstruction was carried out using the modified‐iKM algorithm based on the K–M estimation. The accuracy of each IPD reconstruction was assessed using the *summary()* function and by viewing the output data using *plot()*. After obtaining the IPDs from each study, we labeled the studies according to the comparison performed and generated a pooled IPD for each comparison. We subsequently fitted a pooled survival curve using the *survfit()* function according to time and status as a function of treatment modality. The output of this function was used in *ggplot2()* to generate the survival curve along with p‐values and confidence intervals.

### 2.5 Ethics approval

This study was deemed exempt from Institutional Review Board approval at Hadassah Medical Center, as it was a meta‐analysis of published studies using only de‐identified patient data.

## 3 RESULTS

**FIGURE 1:**
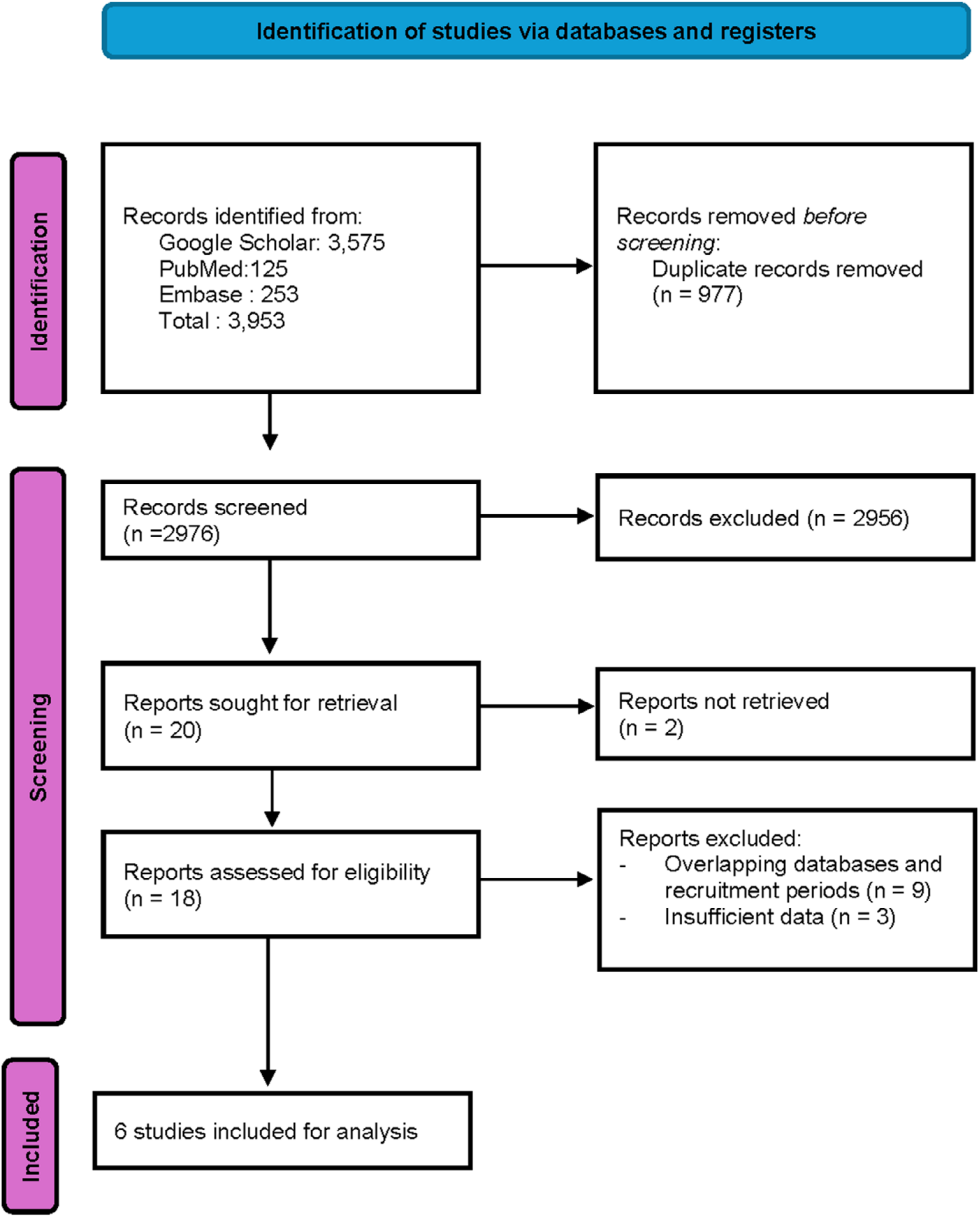
PRISMA flow diagram of study selection. Workflow for identifying and screening eligible studies according to the *Preferred Reporting Items for Systematic Reviews and Meta‐Analyses* (PRISMA) criteria.

A systematic search of the literature identified 3953 publications. After applying the inclusion and exclusion criteria, six cohort studies were included in the final meta‐analysis, comprising five registry‐based studies and one retrospective, multi‐institutional cohort study (Figure 1). Four studies included patients with typical carcinoid (TC), while two studies reported data on atypical carcinoids (ACs). The characteristics of the included studies are summarized in Table 1. Assessment of risk of bias is detailed in Table S3.

**TABLE 1:** Overview of studies included in the meta‐analysis.

Study	Year Published	PSM	Comparison	Database	Years of Patient Inclusion	N Total**	N Lobar**	N Sub‐lobar**	Clinical stage ***	Grade ****	Peripheral vs. Central
Ernani et al.^15^	2022	Yes	Lob vs. Sub	NCDB	2004–2016	218	109	109	T1‐T4N0‐N3M0 Stage 1–3	Atypical	All
Yang et al.^16^	2022	Yes	Lob vs. Sub	SEER	2004–2016	812	406	406	T1‐2N0M0 Stage 1‐2A	Typical	All
Cattoni et al.^17^	2019	No	Lob vs. Sub	Multi institutional *	2000–2015	177	103	74	T1‐3 N0 M0 Stage 1‐2B	Typical	Peripheral
Brown et al.^18^	2018	No	Lob vs. Sub	NCDB	1998–2012	1159	753	406	T1aN0M0 Stage 1A1	Typical	All
Filosso et al.^19^	2018	No	Lob vs. Seg vs. Wed vs. Pneumonectomy	ESTS NETs‐WG	1994–2012	876	679	197	T1‐2aN0M0 Stage 1	Typical	All
Chen et al.^20^	2020	No	Lob vs. Seg vs. Wed	SEER	2001–2016	458	359	99	All	Atypical	All

*Note*: *Swedish Cancer Institute (Seattle, Washington), UC Davis Health (Sacramento, California), Catholic University “Sacred Heart” (Rome, Italy), San Giovanni Battista Hospital (Turin, Italy), University of Insubria–Ospedale di Circolo (Varese, Italy), University of Washington Medical Center (Seattle, Washington), Providence Regional Medical Center (Everett, Washington), and Virginia Mason Hospital and Medical Center (Seattle, Washington). **N represents the actual number of patients included in each study's analysis after application of propensity score matching (PSM), where applicable. ***The studies varied in the applied TNM staging editions. Ernani et al. did not specify the edition. Yang et al. and Chen et al. used the 8th edition, while Cattoni, Brown, and Filosso et al. applied the 7th edition. ****Based on WHO classification: Ernani, Yang, Cattoni, Brown, and Chen did not specify which version was applied. Filosso et al. reported using the 2004 WHO classification along with Travis's histological criteria.

Abbreviations: ESTS NETs‐WG, European Society of Thoracic Surgeons retrospective database of the Neuroendocrine Tumors of the Lung Working Group; Lob, lobectomy; NCDB, National Cancer Database; PSM, propensity score matching; SEER, Surveillance, Epidemiology, and End Results database; Seg, segmentectomy; Sub, sub‐lobar resection; Wed, wedge resection.

A total of 3700 patients were included in the meta‐analysis; 2409 underwent lobectomy and 1291 had sub‐lobar resection. The pooled mean age was 60.2 years (95% CI, 57.7–62.8; *I* ^2^ = 94.1%) in the lobectomy group and 61.9 years (95% CI, 59.6–64.3; *I* ^2^ = 91.1%) in the sub‐lobar resection group (Table 2). The distribution of disease stage, tumor size (T category), nodal status (N category), and metastatic status (M category), as well as the number of lymph nodes examined, lymph node upstaging rates, and completeness of resection (R0/R1), are summarized in Tables S6–S8.

**TABLE 2:** Pooled clinicopathologic characteristics of patients undergoing lobectomy or sub‐lobar resection.

	Lobectomy (*n* = 2409)*		Sub‐lobar Resection (*n* = 1291) *	
	SE (CI)	*I* ^2^	SE	*I* ^2^
Age (years)	60.2 (57.7–62.8)	94.1%	61.9 (59.6–64.3)	91.1%
Male (%)	31.1 (25.8–37.0)	83.1%	24.8 (20.6–29.5)	46.9%
R1 (%) **	2.8 (0–99)	51.5%	7.7 (2.2–23.8)	0%
LN sampling >10 (%) ***	29.1 (0.8–95.3)	96.4%	7.4 (0.01–98)	87.5%
N upstaging (%) ****	6.2 (0.2–64.9)	53.5%	2.2 (0–99)	85.7%

*Note*: *N represents the actual number of patients included in each study's analysis after application of propensity score matching (PSM), where applicable. **Reported in two studies: Ernani et al.^15^ and Brown et al.^18^ ***Reported in two studies: Cattoni et al.^17^ and Brown et al.^18^ ****Reported in two studies: Ernani et al.^15^ and Cattoni et al.^17^

Abbreviations: CI, confidence intervals; *I* ^2^, heterogeneity index; LN, lymph node; SE, standard error.

**FIGURE 2:**
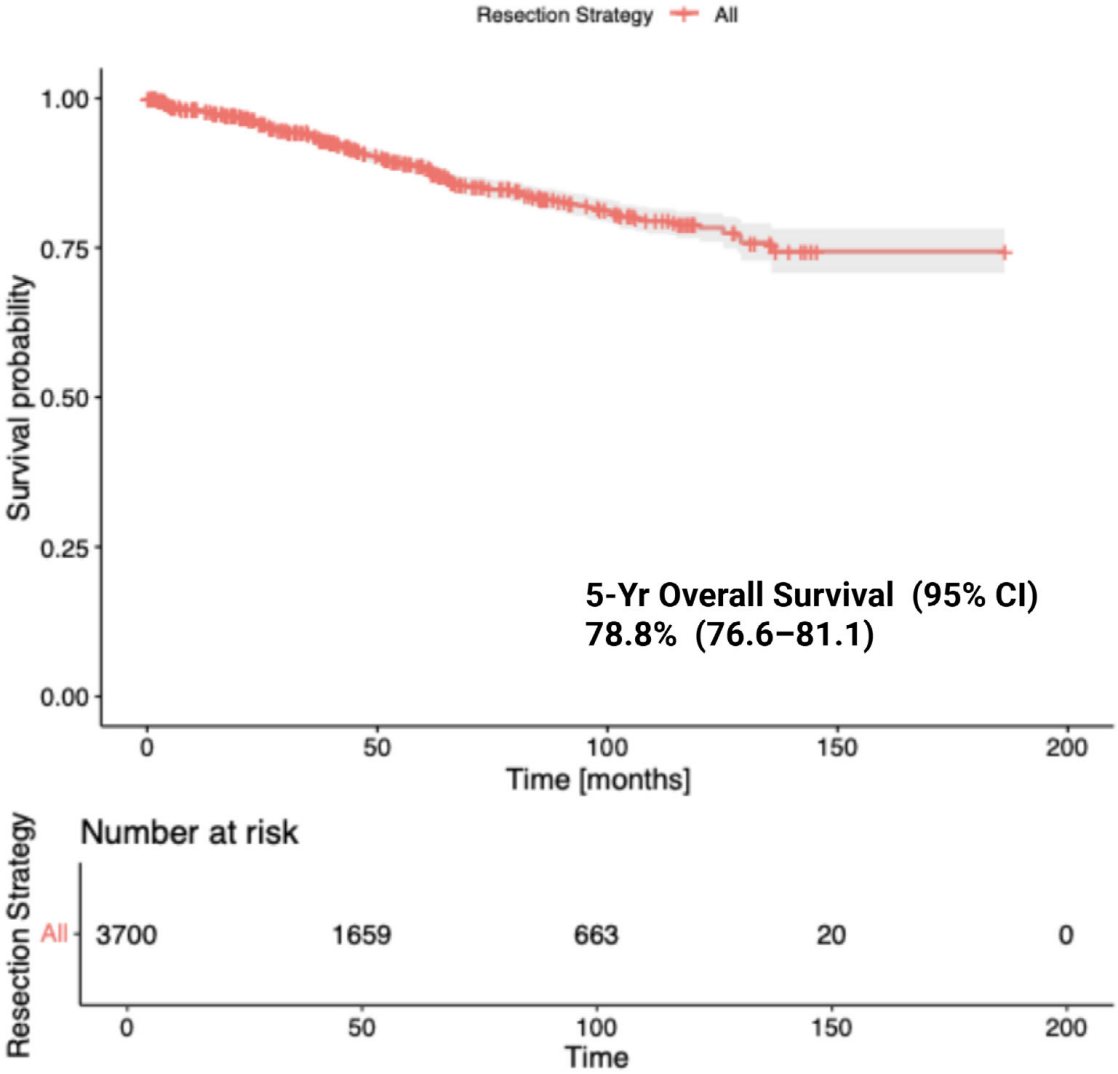
Pooled survival curve. Kaplan–Meier curve generated using reconstructed time‐to‐event data estimated from published Kaplan–Meier curves in all included studies, illustrating overall survival among all patients included in the meta‐analysis.

To estimate the 5‐year overall survival of the entire cohort, we reconstructed a pooled survival plot, using approximated individual time‐to‐event data from the six studies included, encompassing 3700 patients at risk (Figure 2). This dataset was subsequently used for further analyses. The pooled 5‐year overall survival for the entire cohort was 78.8% (95% CI, 76.6–81.1).

### 3.1 Lobectomy vs. sub‐lobar resection

**FIGURE 3:**
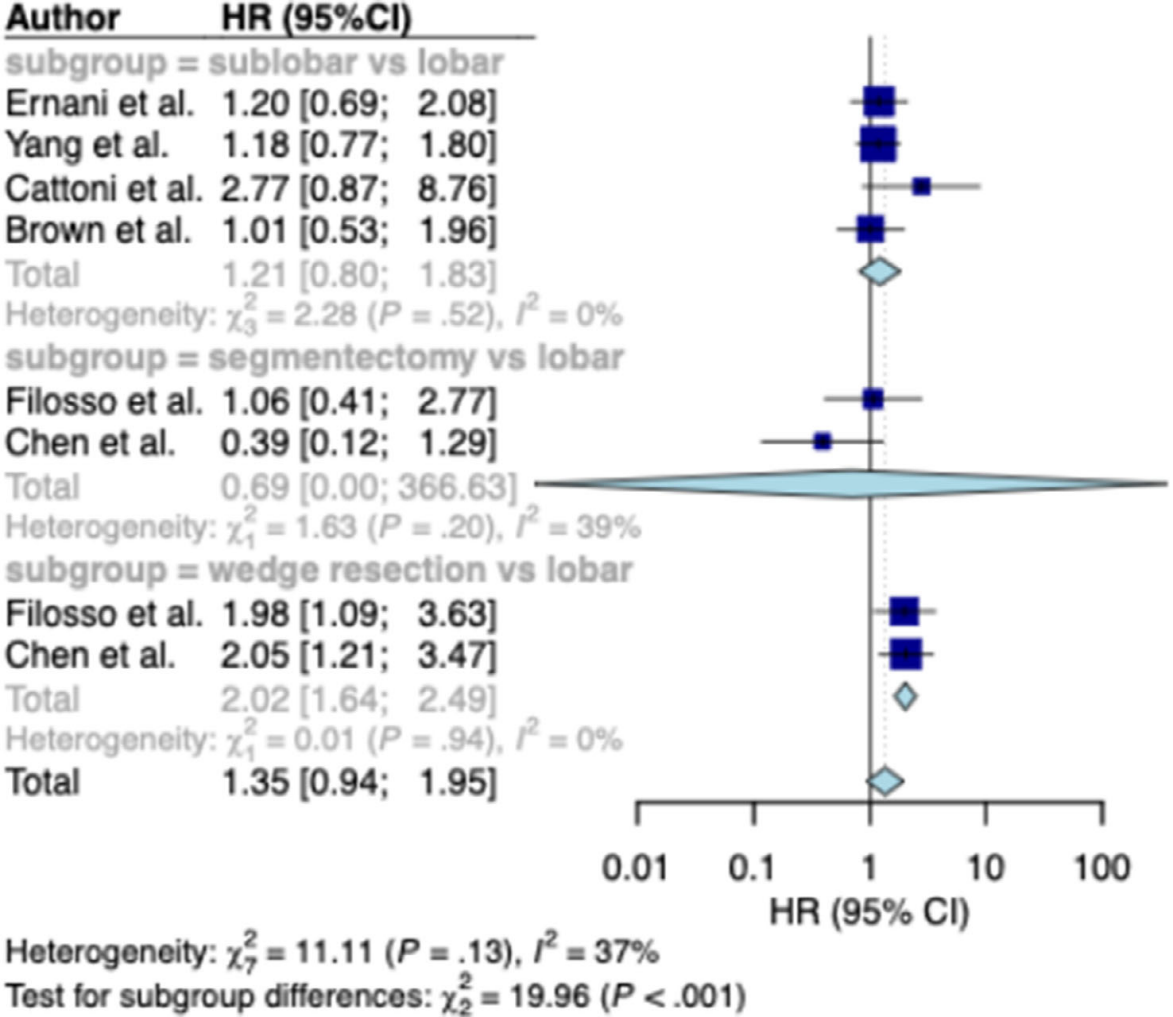
Primary outcome. Meta‐analysis comparing overall survival following lobectomy and sub‐lobar resection, including subgroup analyses by sub‐lobar resection subtype (segmentectomy or wedge resection) compared with lobectomy. HR, hazard ratio; CI, confidence interval.

Overall survival (OS) was evaluated based on data extracted from all six studies. We first compared lobectomy with sub‐lobar resection (*n*_studies = 4; *n* = 1371 and *n* = 995, respectively) (Figure 3, upper panel). No statistically significant difference in OS was observed between the two surgical strategies (HR = 1.21; 95% CI, 0.80–1.83; low certainty of evidence). Inter‐study heterogeneity was negligible (*I* ^2^ = 0%).

**FIGURE 4:**
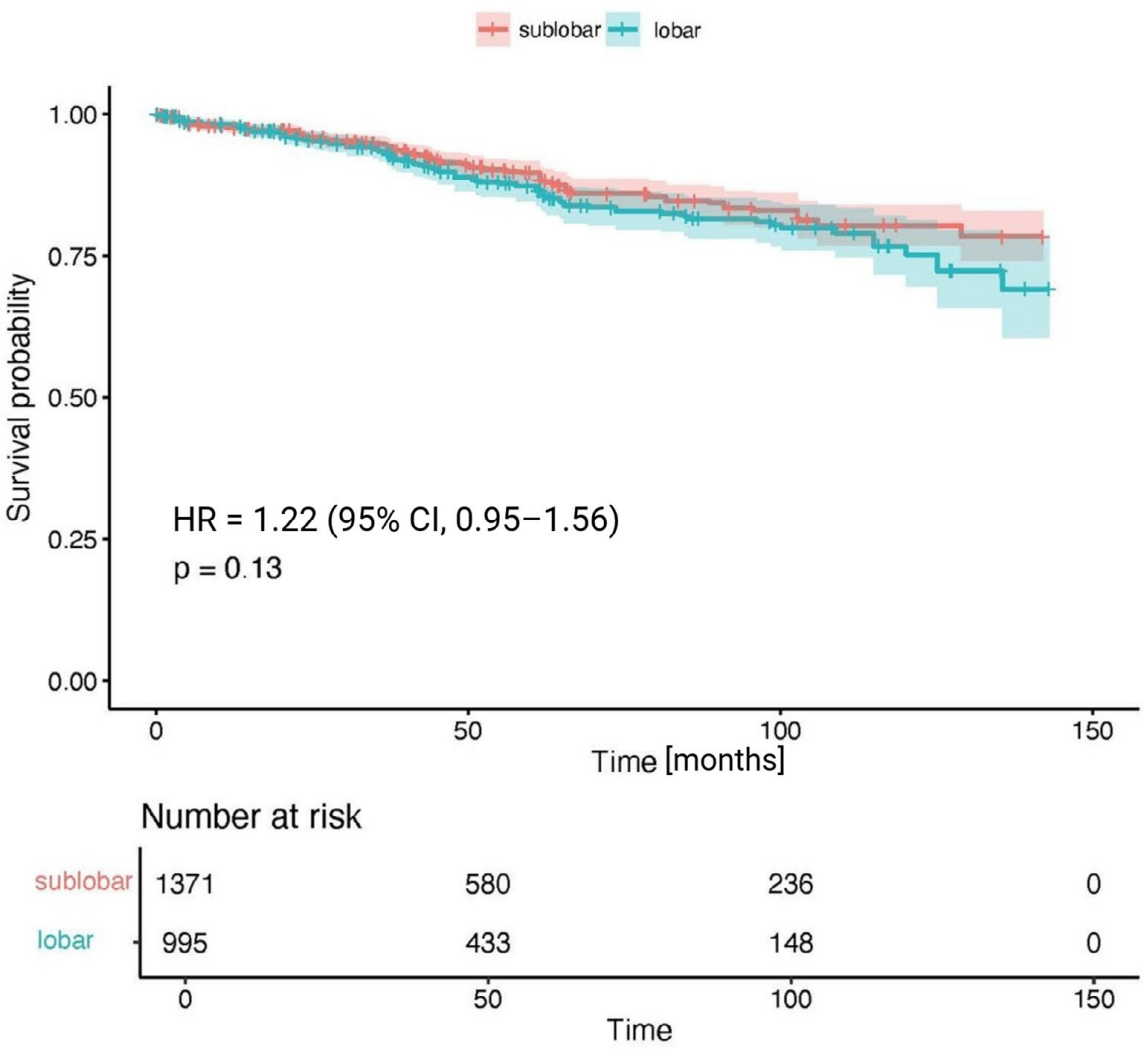
Pooled survival curve. Kaplan–Meier curve generated using reconstructed time‐to‐event data estimated from published Kaplan–Meier curves in four included studies, illustrating overall survival following sub‐lobar versus lobar resection.

To further substantiate these findings, a Cox regression model was applied to the subset of patients from the reconstructed time‐to‐event patient data who underwent either lobectomy or sub‐lobar resection (*n*_studies_ = 4; *n* = 1371 and n = 995, respectively), encompassing 2366 patients at risk. Consistent with the traditional meta‐analysis, no significant difference in OS was detected between the two surgical approaches (HR = 1.22; 95% CI, 0.95–1.56; *p* = 0.13; Figure 4).

### 3.2 Lobectomy vs. segmentectomy and wedge resection

We next compared outcomes of two specific sub‐lobar resection strategies—segmentectomy and wedge resection—with those of lobectomy (*n*_studies_ = 2; *n* = 96 and *n* = 200 vs. *n* = 1038, respectively). No statistically significant difference in overall survival (OS) was observed between segmentectomy and lobectomy (HR = 0.69; 95% CI, 0–366.63; *I* ^2^ = 39%). In contrast, wedge resection showed a trend toward higher mortality compared with lobectomy (HR = 2.02; 95% CI, 1.64–2.49; *I* ^2^ = 0%) (Figure 3, middle and lower panels).

**FIGURE 5:**
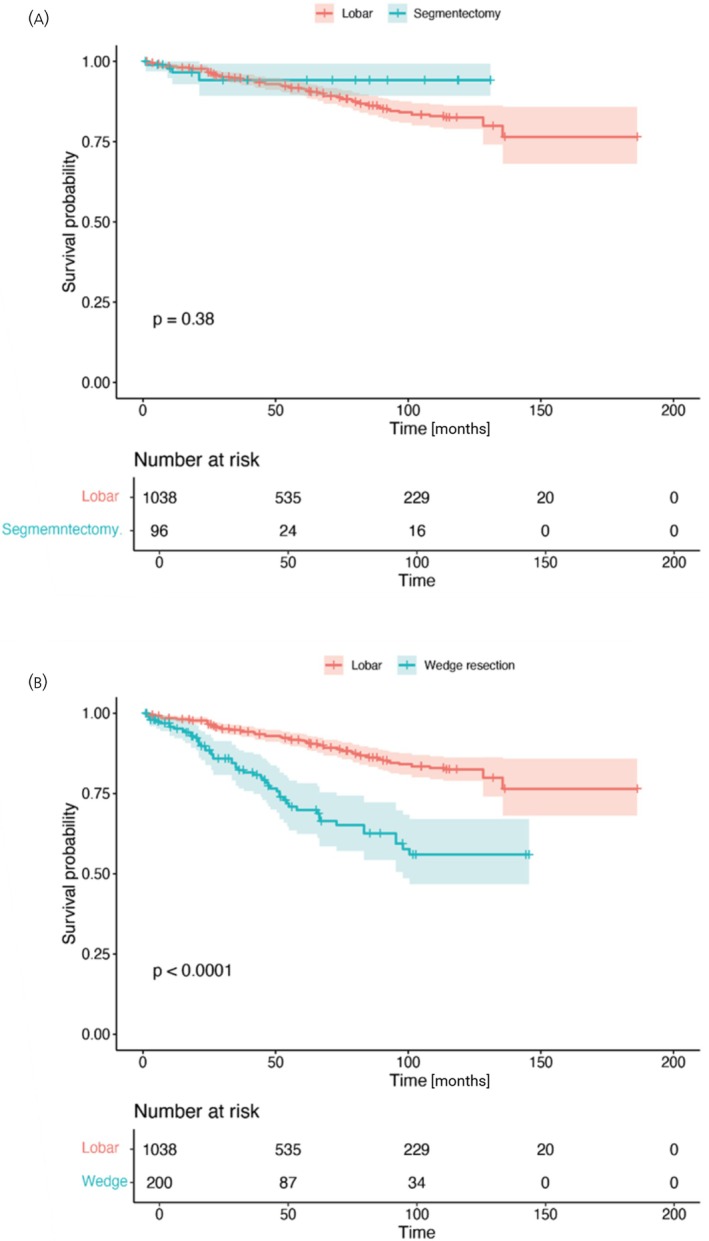
Pooled survival curves. Kaplan–Meier curves generated using reconstructed time‐to‐event data estimated from published Kaplan–Meier curves in two included studies, illustrating overall survival following segmentectomy (A) and wedge resection (B), compared with lobar resection.

Pooled survival curves for these comparisons supported these observations (Figure 5A, B), demonstrating no difference between segmentectomy and lobectomy (*p* = 0.38) and worse survival following wedge resection relative to lobectomy (*p* < 0.0001).

### 3.3 Lymph node metastasis

Patients with clinically evident lymph node involvement were included in only two studies of atypical carcinoid (AC). Pathologic upstaging was reported in two studies of typical carcinoid (TC) (Table S6). Lymph node sampling was uniformly more extensive in the lobectomy group. The pooled proportion of cases with >10 lymph nodes examined was 29.1% (95% CI, 0.8–95.3) in lobectomy and 7.4% (95% CI, 0.01–98) in sub‐lobar resection (Table 2). Correspondingly, clinical‐to‐pathologic nodal upstaging occurred in 6.2% (95% CI, 0.2–64.9) of lobectomy cases and 2.2% (95% CI, 0–99) of sub‐lobar resections (Table 2).

### 3.4 Sensitivity analyses

A leave‐one‐out sensitivity analysis was performed to assess whether the primary outcome was influenced by inter‐study heterogeneity. The recalculated pooled hazard ratios (HRs) for each subgroup are presented in Table S4. Excluding individual studies did not materially alter the overall effect estimate (HR = 1.21; 95% CI, 0.91–1.62; *I* ^2^ = 0%).

## 4 DISCUSSION

We present the first systematic review and meta‐analysis evaluating the effect of surgical strategy on overall survival (OS) in patients with LNETs. Using inverse‐variance meta‐analysis and Cox regression on time‐to‐event data, our analyses suggest that sub‐lobar resection may be associated with OS comparable to lobectomy in both typical carcinoid (TC) and atypical carcinoid (AC). Within the sub‐lobar group, segmentectomy appeared to yield OS similar to lobectomy, whereas wedge resection was associated with a possible trend toward reduced survival.

Current clinical guidelines for the surgical management of lung neuroendocrine tumors (LNETs) are heterogeneous, relying largely on retrospective evidence with conflicting results and, in part, on adaptations from non‐small cell lung cancer (NSCLC) protocols. Organizations such as the European Society for Medical Oncology (ESMO, 2021)^4^ and the European Neuroendocrine Tumor Society (ENETS, 2015) favor lobectomy over segmentectomy and note that wedge resection is associated with increased recurrence—particularly in N‐positive TC or intermediate‐grade AC tumors.^4^, ^21^ In contrast, the National Comprehensive Cancer Network (NCCN, 2021) and the North American Neuroendocrine Tumor Society (NANETS, 2020) endorse both lobectomy and segmentectomy as appropriate options.^22^, ^23^ Furthermore, in 2020, the Commonwealth Neuroendocrine Tumour Research Collaboration (CommNETs) and NANETS jointly updated the 2015 ENETS Expert Consensus Guidelines, suggesting that sub‐lobar resection may be an acceptable approach for peripheral TCs <2 cm in size, provided complete resection is achievable.^24^

Lymph node involvement is not uncommon in LNETs, occurring in 10 to 15% of patients with TC and 30 to 60% of those with AC.^7^ In this analysis, lymph node assessment was less comprehensive in sub‐lobar resections; nevertheless, when performed, a substantial rate of nodal upstaging was identified on histopathological examination. Because nodal upstaging is associated with inferior overall survival and higher recurrence rates,^15^, ^18^, ^20^, ^25^, ^26^ adequate lymph node assessment should always be performed, irrespective of resection extent, to ensure accurate staging.

Given that the vast majority (>2945 cases, approximately 80%) of LNET resections included in our meta‐analysis were performed for clinical stage I disease (Table S6), and that segmentectomy was associated with outcomes comparable to lobectomy even in AC, our findings suggest that lung‐preserving surgery may be a reasonable option for selected patients with clinical stage I (T1‐2aN0M0) LNETs, provided that adequate resection margins and thorough lymph node assessment are ensured. This approach is consistent with current guidelines for the treatment of non–small‐cell lung cancer (NSCLC), a considerably more aggressive malignancy.^5^, ^6^

Among studies that compared sub‐lobar resection subtypes (segmentectomy or wedge resection) with lobectomy (*n*_studies_ = 2), no difference in overall survival (OS) was observed between segmentectomy and lobectomy, whereas wedge resection demonstrated a trend toward higher mortality compared with lobectomy. However, selection bias and surgeon‐related factors cannot be excluded as potential confounders, as none of these studies employed propensity score–matched analyses. Filosso et al. reported that patients who underwent wedge resection were significantly older and had a higher prevalence of prior malignancy compared with those in the lobectomy group, whereas Chen et al. did not provide baseline characteristics stratified by surgical subgroups. Notably, studies comparing wedge resection and segmentectomy using propensity matching—including the largest analysis by Qi et al.,^27^ encompassing 706 patients—have demonstrated comparable outcomes between the two sub‐lobar resection subtypes.^28^, ^29^ Finally, given that in non–small cell lung cancer (NSCLC), wedge resection has been shown to yield outcomes comparable to segmentectomy for tumors <2 cm,^5^ it is possible that a similar approach could be considered in selected patients with lung neuroendocrine tumors (LNETs) <2 cm, provided that strict quality standards for wedge resection are met.^30^

Of the included studies, only Cattoni et al.^17^ reported recurrence‐free survival (RFS), with results indicating no significant difference between lobectomy and sub‐lobar resection (HR = 1.82; 95% CI, 0.54–6.13).

Beyond its potential oncologic equivalence in selected cases, sub‐lobar resection may confer the additional advantage of improved postoperative pulmonary function, as reflected by higher forced expiratory volume in one second (FEV₁) and forced vital capacity (FVC).^31^

In light of our findings indicating that sub‐lobar resection may be associated with outcomes similar to lobectomy in selected patients with clinical stage I disease, it remains important to acknowledge the complexity of standardized decision‐making in LNETs. Their rarity, the frequent lack of adequate tissue for pathological diagnosis prior to surgery, the biological heterogeneity—including a recently proposed category of LNETs with carcinoid morphology but proliferative indices exceeding those of conventional AC^32^ —and clinical variability among patients all underscore the need for evaluation in specialized centers with expertise in LNETs, where surgical strategies can be tailored to ensure complete resection, lymph node assessment, and careful consideration of comorbidities.

Eligibility for quantitative analysis was limited by the reliance of many published studies on large databases, particularly SEER and NCDB, necessitating exclusion of overlapping cohorts. Recent single‐center studies by Bertolaccini et al.^33^ and Hojerat et al.^34^ offer complementary evidence from independent patient populations and similarly reported no clear survival difference between lobar and sub‐lobar resections in early‐stage LNETs. Our study has several limitations. We applied the *Grading of Recommendations Assessment, Development and Evaluation* (GRADE) framework to assess the quality of evidence and strength of recommendations. Based on this assessment, the certainty of evidence for the primary outcome was rated as **low**, indicating limited confidence in the effect estimate and the possibility that the true effect may differ substantially. All included studies were retrospective cohort analyses, which inherently carry a risk of selection bias and therefore contributed to the downgrading of evidence certainty. Patients selected for sub‐lobar resection may have had less aggressive radiologic features or, conversely, may have been considered unsuitable for lobectomy due to advanced age or significant comorbidities. The absence of granular data limited our ability to fully adjust for these potential confounders. Nonetheless, two of the six studies included in the final analysis employed propensity score matching, which helped mitigate baseline imbalances between surgical groups. In addition, we used two independent statistical approaches to evaluate the primary outcome, and the *I* ^2^ statistic for comparisons between sub‐lobar and lobar resection was 0%, indicating negligible interstudy heterogeneity. Despite these strengths, further high‐quality, prospective studies are warranted to validate these findings and better inform clinical decision‐making.

Variability in pathologic classification and staging criteria across studies was an additional limitation. Different WHO classification versions for grading (TC vs. AC) and different TNM editions were used (Table 1). Nevertheless, we attempted to harmonize data when tumor size (in centimeters) was reported, which improved consistency.

Sub‐lobar resection is technically feasible primarily for peripheral tumors. Among the included studies, only Cattoni et al.^17^ restricted their cohort to peripheral lesions, whereas the others did not provide data regarding tumor location (central vs. peripheral), potentially limiting the generalizability and applicability of our findings.

Additionally, the impact of resection strategy may differ across histologic subtypes (typical vs. atypical carcinoid). However, we were unable to examine this aspect in depth due to methodological constraints. Only two studies included patients with atypical carcinoid (AC). Of these, Chen et al. reported separate hazard ratios for segmentectomy and wedge resection relative to lobectomy. Combining these data would have required double‐counting the lobectomy group, thereby introducing statistical bias.

Furthermore, two studies reported outcomes for segmentectomy and wedge resection separately in comparison with lobar resection, precluding the use of a leave‐one‐out sensitivity analysis to evaluate potential influencer bias. Nevertheless, we generated a pooled Kaplan–Meier curve, which supported the robustness of our findings.

## 5 CONCLUSIONS

In conclusion, this first systematic review and meta‐analysis evaluating surgical strategy and overall survival (OS) in lung neuroendocrine tumors (LNETs) suggests that sub‐lobar and lobar resections may be associated with comparable OS in patients with clinical stage I disease. We underscore the critical importance of adequate lymph node assessment regardless of surgical strategy and highlight individualized treatment at specialized multidisciplinary LNETs centers as key to achieving optimal outcomes. Importantly, prospective, multicenter studies are critically needed to validate these findings and better define surgical standards for this rare tumor entity.

## AUTHOR CONTRIBUTIONS

**Gal Aviel**: Conceptualization (equal); formal analysis; methodology; software; writing – original draft; preparation. **Ranin Hojerat**: Investigation; data curation; writing – original draft; writing – review and editing; visualization. **Islam Idais**: Investigation. **Bruria Hirsh‐Raccah**: methodology. **Simona Grozinsky‐Glasberg**: conceptualization (supporting); writing – review and editing. **Anat Bel Ange**: conceptualization (supporting). **Oz M. Shapira**: conceptualization (supporting). **Amit Korach**: conceptualization (supporting). **Uzi Izhar**: conceptualization (supporting); validation. **Ori Wald**: conceptualization (equal); project administration; supervision; validation; writing – review and editing.

## Supporting information


**TABLE S1.** PICO table and objective.
**TABLE S2.** Search Strategy.
**TABLE S3.** The Newcastle‐Ottawa Scale (NOS) for assessing the quality of studies in meta‐analyses.
**TABLE S4.** Sensitivity analysis: leaving‐one‐out method.
**TABLE S5.** GRADE score for Primary Outcome.
**TABLE S6.** Baseline Characteristics of Included Study Cohorts.
**TABLE S7.** Lobectomy Subgroups: Baseline Characteristics.
**TABLE S8.** Sublobar Subgroups: Baseline Characteristics.

## Data Availability

The data that supports the findings of this study are available in the supplementary material of this article.
