# Supplementary material for: The lesser the better? A systematic review and meta‐analysis of resection strategy in lung neuroendocrine tumors
Source: J Neuroendocrinol. 2026 Apr 1;38(4):e70176. doi: 10.1111/jne.70176 (PMC13043326; doi:10.1111/jne.70176)
Supplement: Supplementary file 1 — TABLE S1. PICO table and objective. TABLE S2. Search Strategy. TABLE S3. The Newcastle‐Ottawa Scale (NOS) for assessing the quality of studies in meta‐analyses. TABLE S4. Sensitivity analysis: leaving‐one‐out method. TABLE S5. GRADE score for Primary Outcome. TABLE S6. Baseline Characteristics of Included Study Cohorts. TABLE S7. Lobectomy Subgroups: Baseline Characteristics. TABLE S8. Sublobar Subgroups: Baseline Characteristics. [file JNE-38-e70176-s001.docx]

**Appendix 1**

Contents

[Table S1. PICO table and objective 2](#_Toc211625177)

[Table S2. Search Strategy 3](#_Toc211625178)

[Table S3. The Newcastle-Ottawa Scale (NOS) for assessing the quality of studies in meta-analyses 4](#_Toc211625179)

[Table S4. Sensitivity analysis: leaving-one-out method 5](#_Toc211625180)

[Table S5. GRADE score for Primary Outcome 6](#_Toc211625181)

[Table S6. Baseline Characteristics of Included Study Cohorts 7](#_Toc211625182)

[Table S7. Lobectomy Subgroups : Baseline Characteristics 8](#_Toc211625183)

[Table S8. Sublobar Subgroups : Baseline Characteristics 9](#_Toc211625184)

## Table S1. PICO table and objective

Objective: Compare the effect oft he extent of surgical resection on overall survival in patients with pulmonary neuroendocrine tumors.


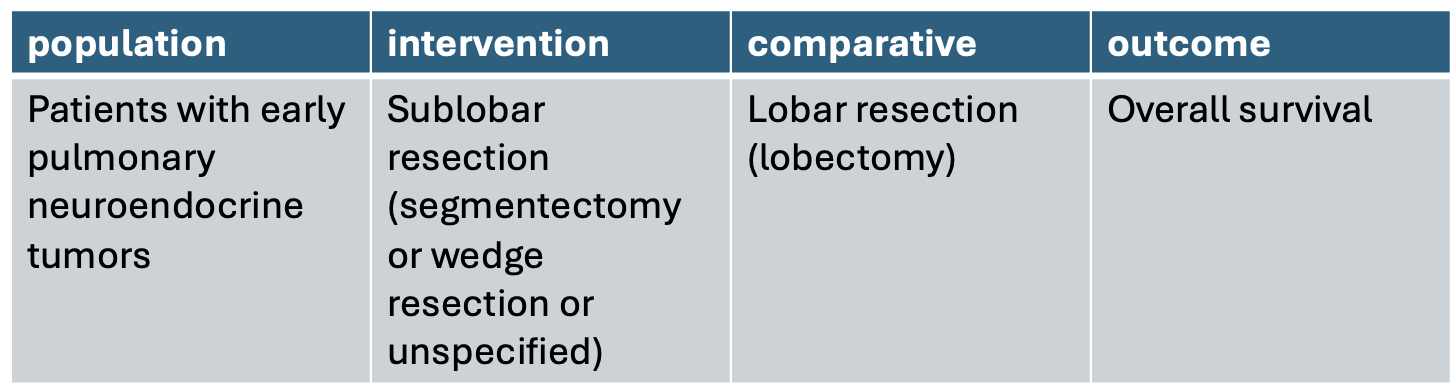


## Table S2. Search Strategy : Database inception to September 26, 2024


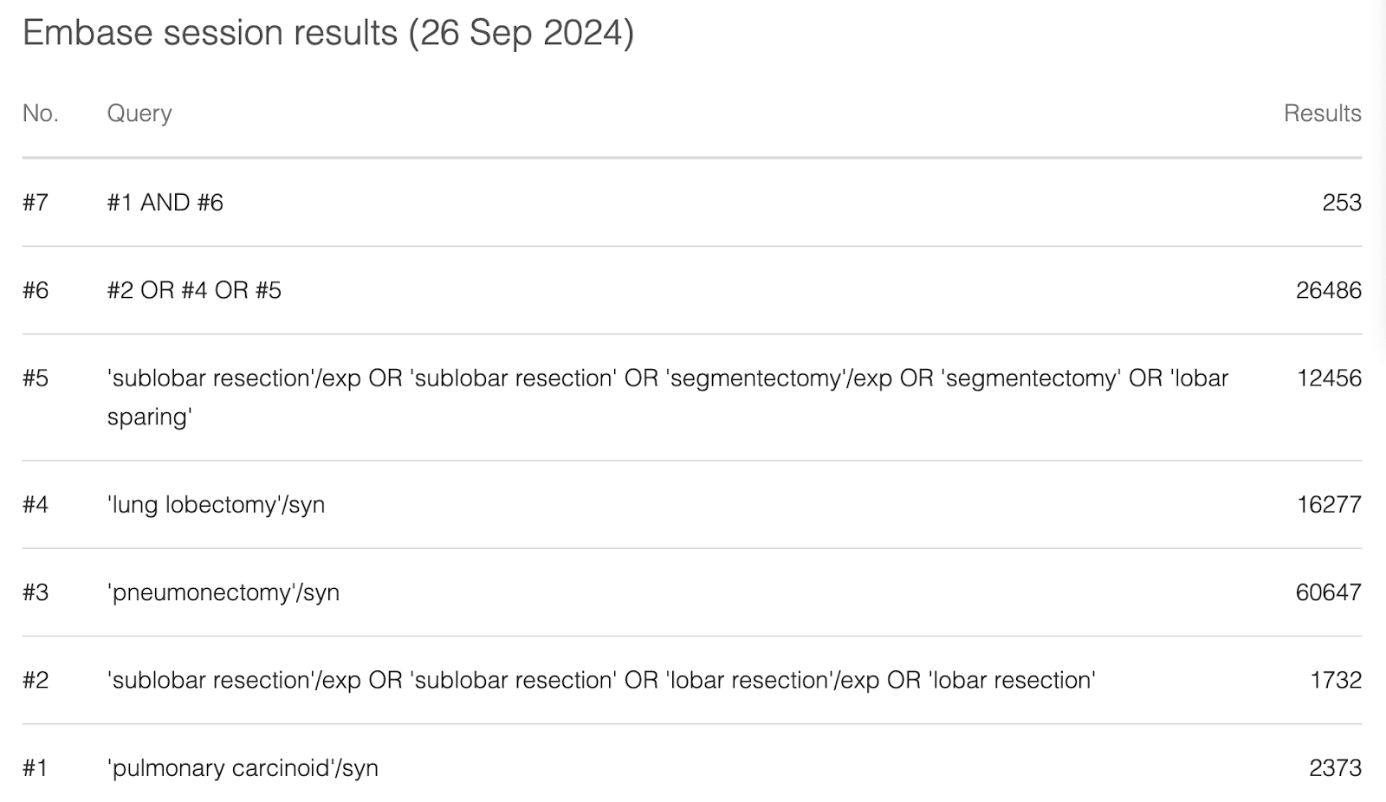


PubMed , Cochrane Library and Google Scholar Search Strategy

("pulmonary neuroendocrine" OR "pulmonary NET" OR "pulmonary carcinoid" OR "lung carcinoid") AND ("lobar resection" OR "segmentectomy" OR "wedge" OR "parenchymal sparing" OR "lobectomy")

| **Study** | **Selection** | | | | **Comparability** | | **Outcome** | | | **Total Quality Score** |
| --- | --- | --- | --- | --- | --- | --- | --- | --- | --- | --- |
|  | **Representativeness of exposed cohort** | **Selection of the non-exposed cohort** | **Ascertainment of exposure** | **Demonstration that outcome of interest was not present at start of study** | **Controls for severity** | **Controls for additional risk factors** | **Assessment of outcome** | **Was follow-up long enough?** | **Adequacy of follow-up** |  |
| Ernani et al. ^20^ | 1 | 1 | 1 | 1 | 1 | 1 | 1 | 1 | 1 | 9 |
| Yang et al. ^31^ | 1 | 1 | 1 | 1 | 1 | 1 | 1 | 1 | 1 | 9 |
| Cattoni et al. ^32^ | 1 | 1 | 1 | 1 | 1 | 1 | 1 | 1 | 1 | 9 |
| Brown et al. ^21^ | 1 | 1 | 1 | 1 | 0 | 0 | 1 | 1 | 1 | 7 |
| Filosso et al. ^33^ | 1 | 1 | 1 | 1 | 0 | 0 | 1 | 1 | 1 | 7 |
| Chen et al. ^22^ | 1 | 1 | 1 | 1 | 0 | 0 | 1 | 1 | 1 | 7 |

## Table S3. The Newcastle-Ottawa Scale (NOS) for assessing the quality of studies in meta-analyses

## Table S4. Sensitivity analysis: leaving-one-out method

## Table S5. GRADE score for Primary Outcome


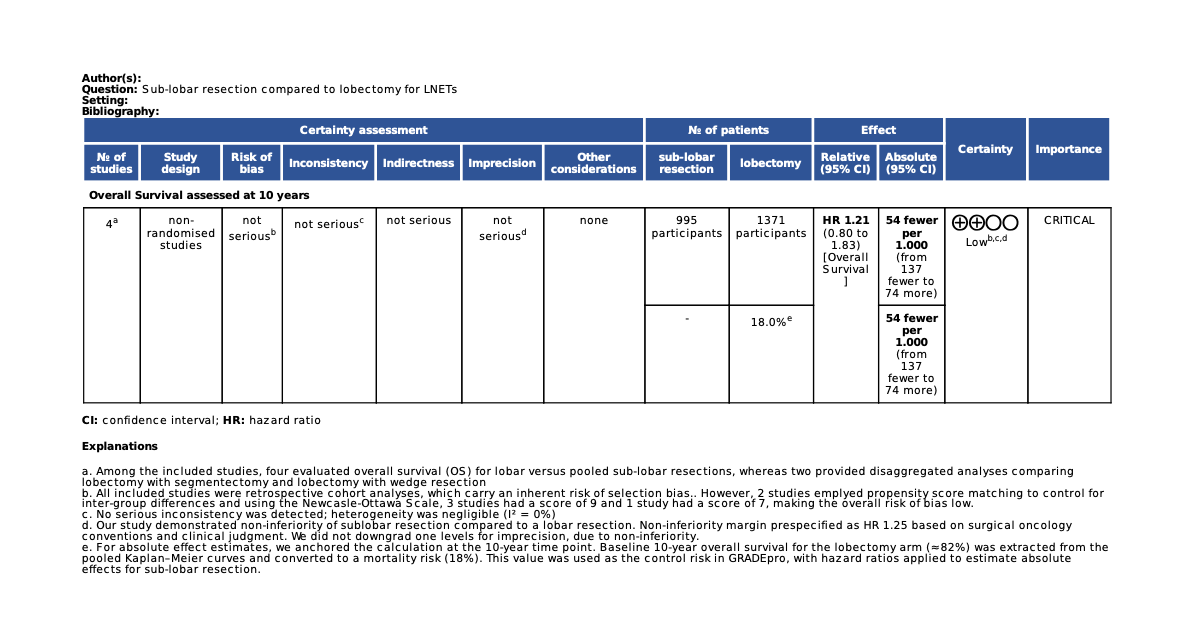


## Table S6. Baseline Characteristics of Included Study Cohorts

| Study | N | Age (years) | Female Sex (N, %) | cT (N, %) | cN (N, %) | Lymph Node (LN) Assessment | Nodal Upstaging (N, %) | R0/R1 (N, %) | Previous malignancy/ another primary tumour (N, %) | Follow up time (months) |
| --- | --- | --- | --- | --- | --- | --- | --- | --- | --- | --- |
| Ernani et al. ^20^ | 669* | (N, %)  ≤65 349 (52.2)  >65 320 (47.8) | 453 (67.7) | T1 491 (73.4)  T2 154 (23.0)  T3–T4 24 (3.6) | 185 (27.6) | ≤10 LN 393 (64.7)  > 10 LN 214 (35.3) | Not reported | R0 637 (95.2)  R1 32 (4.8) | Not reported | Not reported |
| Yang et al. ^31^ | 812 | Mean age ± SD  60.27 ± 13.25 | 584 (71.92) | T1 766 (94)  T2 46 (6)  T3–T4 0 (0) | 0 (0) | Mean ± SD  3.71 ± 4.93 | 0 (0) **** | Not reported | Not reported | Median - 73 |
| Cattoni et al. ^32^ | 177 | Median (IQR)  62 (54 - 70) | 131 (74) | T1 144 ( 81.81)  T2 24 (13.63)  T3–T4 8 (4.54) | 0 (0) | Any LN assessment 132 (74.6) | 13 (7.3) | R0 170 (96.6)  R1 6 (3.4) | 34 (19.2) | Mean - 58.1 |
| Brown et al. ^21^ | 1495** | Median (IQR)  Lobectomy 62 (54–69)  Sublobar 64 (56–71) | 1124 (75.18) | T1 1495 (100)  T2 0 (0)  T3–T4 0 (0) | 0 (0) | None 366 (24.5)  ≤10 LN 802 (53.64)  > 10 LN 250 (16.7)  Not quantified 77 (5.15) | 60 (4) | Not reported | 0, 0 | Median - 35.3 (IQR: 22.4 - 56.0) |
| Filosso et al. ^33^ | 876 | Median (IQR)  60 (47-69) | 569 (65) | T1-T2a 876 (100)  T2b–T4 0 (0) | 0 (0) | Not reported | 0 (0) **** | Not reported | 160 (18) | Not reported |
| Chen et al. ^22^ | 507*** | (N, %)  ≤60 254 (50.1)  61-70 143 (28.2)  >70 110 (21.7) | 329 (64.89) | T1 251 (51.43)  T2 145 (29.71)  T3–T4 92 (5.94 ) | 167 (33.5) | Any LN assessment 423 (83.6) | Not reported | Not reported | 0 , 0 | Mean - 53.22 |

## Table S7. Lobectomy Subgroups : Baseline Characteristics

| Study | N | Age (years) | Female Sex (N, %) | cT (N, %) | cN (N, %) | Lymph Node (LN) Assessment | Nodal Upstaging (N, %) | R0/R1 (N, %) | Previous malignancy/ another primary tumour (N, %) |
| --- | --- | --- | --- | --- | --- | --- | --- | --- | --- |
| Ernani et al. ^20^ | 560 * | (N, %)  ≤65 298 (53.2)  >65 262 (46.8) | 369 ,(65.9) | T1 396 (70.7)  T2 145 (25.9)  T3-T4 19 (3.4) | 164 (29.3) | ≤10 LN 309 (60.6)  >10 LN 201 (39.4) | Not reported | R0 537 (95.9)  R1 23 (4.1) | Not reported |
| Yang et al. ^31^ | 406 | Mean ± SD  60.66 ± 14.05 | 292 (71.92) | T1 381 (93.84)  T2 25 (6.16)  T3–T4 0 (0 ) | 0 (0) | Mean ± SD 3.84 ± 3.98 | 0 (0) **** | Not reported | Not reported |
| Cattoni et al. ^32^ | 103 | Median (IQR)  63 (54-70) | 73 (70.87) | T1 78 (76)  T2 22 (22)  T3-T4 2 (2) | 0 (0) | Any LN assessment 93 (90.3) | 9 (8.74) | R0 101 (99)  R1 1 (1) | 14 (14) |
| Brown et al. ^21^ | 959 ** | Median (IQR)  62 (54–69) | 703 (73.3) | T1 959 (100)  T2 0 (0)  T3-T4 0 (0) | 0 (0) | None 70 (7.3)  1–9 LNs 614 (64.0)  > 10 LNs 223 (23.3)  Not quantified 52 (5.4) | 55 (5.7) | Not reported | 0, 0 |
| Filosso et al. ^33^ | 679 | Median (IQR)  58 (45-67) | 428 (63) | T1-T2 679 (100)  T3-T4 0 (0) | 0 (0) | Not reported | 0 (0) **** | Not reported | 98 (14) |
| Chen et al. ^22^ | 359 *** |  |  |  |  |  |  |  |  |

## Table S8. Sublobar Subgroups : Baseline Characteristics

| Study | N | Age (years) | Female Sex (N, %) | cT (N, %) | cN (N, %) | Lymph Node (LN) Assessment | Nodal Upstaging (N, %) | R0/R1 (N, %) | Previous malignancy/ another primary tumour (N, %) |
| --- | --- | --- | --- | --- | --- | --- | --- | --- | --- |
| Ernani et al. ^20^ | 109 | (N, %)  ≤65 51 (46.8)  >65 58 (53.2) | 84 (77.1) | T1 95 (87.2)  T2 9 (8.3)  T3–T4 5 (4.6) | 21 (19.3) | ≤10 84 (86.6)  >10 13 (13.4) | Not reported | R0 100 (91.7)  R1 9 (8.3) | Not reported |
| Yang et al. ^31^ | 406 | Mean age ± SD  59.95 ± 12.39 | 292 (71.92) | T1 385 (94.83)  T2 21 (5.17)  T3–T4 0 (0 ) | 0 (0) | Mean ± SD 3.57 ± 5.73 | 0 (0) **** | Not reported | Not reported |
| Cattoni et al. ^32^ | 74 | Median (IQR)  62 (54-71) | 58 (78.37) | T1 66 (89 )  T2 2 (3)  T3-T4 6 (8) | 0 (0) | Any LN assessment 39 (52.7) | 4 (5.4) | R0 69 (93)  R1 5 (7) | 20 (27) |
| Brown et al. ^21^ | 536 ** | Median (IQR)  64 (56–71) | 421 (78.5) | T1 536 (100 )  T2 0 (0 )  T3-T4 0 (0 ) | 0 (0) | None 294 (56.5)  1–9 LN 180 (34.6)  > 10 LN 25 (4.8)  Not quantified 21 (4) | 5 (0.93) | Not reported | 0 (0) |
| Filosso et al. ^33^ |  |  |  |  |  |  |  |  |  |
| Segmentectomy | 75 | Median (IQR)  63 (55.5–70.5) | 50 (67) | T1 - T2 75 (100 )  T3-T4 0 (0 ) | 0 (0) | Not reported | 0 (0) **** | Not reported | 17 (23) |
| Wedge Resection | 122 | Median (IQR)  67 (58-74) | 91 (75) | T1 - T2a 122 (100 )  T3-T4 0 (0 ) | 0 (0) | Not reported | 0 (0) **** | Not reported | 45 (37) |
| Chen et al. ^22^ |  |  |  |  |  |  |  |  |  |
| Segmentectomy | 21 *** |  |  |  |  |  |  |  |  |
| Wedge Resection | 78 *** |  |  |  |  |  |  |  |  |

Values are reported as mean ± standard deviation (SD), median with interquartile range (IQR), or number (percentage), as presented in the original studies.

^*^ Baseline characteristics were reported for the entire cohort only; no subgroup characteristics were provided following propensity score matching, which was used for the survival analyses (Kaplan-Meier and multivariable Cox models)

^**^ The total number of patients reported in the baseline characteristics table differs from the numbers at risk displayed in the Kaplan–Meier curves. The original study did not provide an explanation for this discrepancy.

^***^ Baseline characteristics were reported for 507 patients; however, only 458 patients were included in the cox multivariable survival and K-M analyses after exclusion of cases with missing data. In addition, baseline characteristics were not stratified by surgical subgroups.

^****^ Only pathologically confirmed N0 patients were included in this study
